# Metal-Specificity Divergence between Metallothioneins of *Nerita peloronta* (Neritimorpha, Gastropoda) Sets the Starting Point for a Novel Chemical MT Classification Proposal

**DOI:** 10.3390/ijms222313114

**Published:** 2021-12-04

**Authors:** Mario García-Risco, Sara Calatayud, Veronika Pedrini-Martha, Ricard Albalat, Reinhard Dallinger, Òscar Palacios, Mercè Capdevila

**Affiliations:** 1 Departament de Química, Facultat de Ciències, Universitat Autònoma de Barcelona, E-08193 Cerdanyola del Vallès, Spain; Mario.GarciaRisco@uab.cat (M.G.-R.); oscar.palacios@uab.cat (Ò.P.); 2 Departament de Genètica, Microbiologia i Estadística and Institut de Recerca de la Biodiversitat (IRBio), Facultat de Biologia, Universitat de Barcelona, Av. Diagonal 643, E-08028 Barcelona, Spain; sarak09@hotmail.com (S.C.); ralbalat@ub.edu (R.A.); 3 Institute of Zoology and Center of Molecular Biosciences, University of Innsbruck, Technikerstraße 25, A-6020 Innsbruck, Austria; veronika.pedrini-martha@uibk.ac.at

**Keywords:** metallothioneins, Cd-selective, Neritimorpha, biochemical classification

## Abstract

Metallothioneins’ (MTs) biological function has been a matter of debate since their discovery. The importance to categorize these cysteine-rich proteins with high coordinating capacity into a specific group led to numerous classification proposals. We proposed a classification based on their metal-binding abilities, gradually sorting them from those with high selectivity towards Zn/Cd to those that are Cu-specific. However, the study of the NpeMT1 and NpeMT2isoforms of *Nerita peloronta*, has put a new perspective on this classification. *N. peloronta* has been chosen as a representative mollusk to elucidate the metal-binding abilities of Neritimorpha MTs, an order without any MTs characterized recently. Both isoforms have been recombinantly synthesized in cultures supplemented with Zn^II^, Cd^II^, or Cu^II^, and the purified metal–MT complexes have been thoroughly characterized by spectroscopic and spectrometric methods, leading to results that confirmed that Neritimorpha share Cd-selective MTs with Caenogastropoda and Heterobranchia, solving a so far unresolved question. NpeMTs show high coordinating preferences towards divalent metal ions, although one of them (NpeMT1) shares features with the so-called genuine Zn-thioneins, while the other (NpeMT2) exhibits a higher preference for Cd. The dissimilarities between the two isoforms let a window open to a new proposal of chemical MT classification.

## 1. Introduction

Metallothioneins (MTs) form a family of metalloproteins characterized by their mostly low molecular weight and high content of cysteine (Cys) residues, which confer on them the ability of coordinating heavy metal ions [1,2]. Thanks to this ability, MTs and their isoforms can exert different biological functions, generally related with metal homeostasis and detoxification [3]. Highly significant is their role in scavenging cadmium ions [4,5]. Due to its chemical resemblance to the essential zinc, Cd^II^ competes with the Zn^II^ ions present in several protein metal-binding sites [6]. This fact has led evolutionary ecologists to associate different situations of metal availability with micro-evolutionary and population-specific adaptations of these proteins and vice versa [7,8,9]. A holistic view suggested, for example, that differences in the availability of Cd^II^ in worldwide habitats was a factor that influenced MT evolution in mollusks [10,11] and other animals [12], while the diversification in the kind and degree of metal selectivity in MTs might have had implications on the adaptation, and subsequent settlement, of marine lineages to terrestrial and freshwater environments with different metal availabilities [13,14]. This means that the metal preference of the current MTs is the result of the ecological and physiological boundary conditions under which different organisms have had to deal with, and thereby, that the evolution of MTs with new metal preferences would be related to colonization events of the species towards novel habitats.

The metal preference of MTs is determined by the disposition of the Cys residues within the protein chain, as well as by the nature and spatial positioning of other non-coordinative amino acids such as asparagine (Asp), lysine (Lys), and other residues contiguous to Cys [15]. To aid the understanding of MTs’ metal-binding mechanisms, some criteria were established to classify them according to their chemical binding preferences [16], in addition to their evolved metal-binding specificities along classic taxonomic and phylogenetic relationships [17]. Thus, MTs may either display a Cd/Zn-thionein character, if they yield unique homometallic metal–MT species when produced in divalent metals surplus, or a Cu-thionein character, when they render well-structured homometallic Cu^I^ species under Cu^II^ supplementation [18]. As more and more MTs were explored and characterized, this dichotomic criterion was updated to a stepwise gradation between these two extreme binding preferences [19], meaning that not all MTs strictly comply a strict division between these two states.

The evolution of MTs in many major mollusk clades was driven by Cd^II^ bioavailability [10,11]. Accordingly, a number of mollusk MTs were chemically characterized and sorted along their specific metal-binding preferences [20], in addition to analyses of their metal association and metal-related functions in in vivo [21]. In the present study, we have focused on the MTs of the gastropod *Nerita peloronta* (commonly called the “bleeding tooth”), a species of sea snail belonging to the order Neritimorpha, which is the sister clade to Caenogastropoda and Heterobranchia [22]. Through evolution, some lineages of this clade have been able to colonize terrestrial and freshwater realms [23,24]. *N. peloronta* lives in the upper intertidal surf zone of tropical Eastern Pacific and Atlantic regions [25,26], being continuously exposed to the impact of tidal splash-water and other harsh conditions [27]. Thus, the snail exhibits a high degree of stress resistance to site-specific environmental conditions [28]. Diverse species of the genus *Nerita* have been shown to accumulate heavy metals in their shells and soft tissues, which qualifies them as biological indicators for marine metal pollution [29,30,31].

Like all Neritimorpha species considered to date, *N. peloronta* possesses two MTs, namely NpeMT1 and NpeMT2 [10,11], which may have originated by a lineage-specific gene duplication [10]. The two NpeMTs show the typical modular organization of gastropod MTs, made of a gastropod-specific amino-terminal β3 domain combined with a carboxyl-terminal β1 domain [11]. In depth characterization of the biochemical properties and metal-binding features of these β3/β1 MTs of *N. peloronta*, as well as of the β3 domain of NpeMT2 (β3NpeMT2), offers the opportunity to expand the so-far available knowledge on metal-selectivity of snail MTs to an order of gastropods that has been poorly studied so far.

For that reason, NpeMT1, NpeMT2, and the β3 domain of NpeMT2 have been recombinantly synthesized in and purified from Zn^II^-, Cd^II^-, or Cu^II^-supplemented *E. coli* cultures. The metal-binding behavior of the two MTs plus that of the β3 domain was assessed by means of both spectroscopic techniques (ICP–AES, CD, and UV-vis) and mass spectrometry (ESI–MS). Our results confirm the metal-selective character of NpeMTs, revealing novel metal-specific features that led to an update of our proposed metal binding preference classification of these proteins, giving rise to a discussion about the biological relevance of some snail MTs for the binding and handling of Cd^II^ and Zn^II^ ions.

## 2. Results and Discussion

### 2.1. Heterologous Expression and Production of Metal-NpeMT Complexes

**Figure 1:**
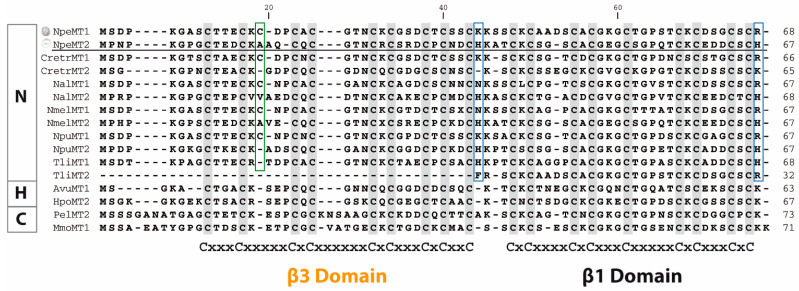
Amino acid alignment of Neritimorpha (N) MTs including those of *Nerita peloronta* (NpeMT1 and NpeMT2), *Clithon retropictum* (CretrMT1 and CretrMT2), *Nerita albicilla* (NalMT1 and NalMT2), *Nerita melanotragus* (NmelMT1 and NmelMT2), *Neritina pulligera* (NpuMT1 and NpuMT2), and *Titiscania limacina* (TliMT1 and TliMT2). Neritimorpha MTs are compared and aligned with selected MTs of Heterobranchia (H) and Caenogastropoda (C): *Arion vulgaris* (AvuMT1), *Helix pomatia* (HpoMT2), *Pomatias elegans* (PelMT2), and *Marseniopsis mollis* (MmoMT1). Conserved cysteines are highlighted with a grey background. Extra cysteines in the MT1 of the Neritimorpha are highlighted in a green box and extra histidines in the MT2s are in a blue box. Cysteine arrangements in the respective β3 and β1 domains are shown below the alignment.

To characterize the biochemical properties and metal-binding features of NpeMT1 and NpeMT2, both proteins were expressed as GST–MT fusion proteins in *E. coli* BL21 cultures supplemented with ZnCl_2_, CdCl_2_, or CuSO_4_. The amino acid sequences of the expressed NpeMT1 and NpeMT2 are 57.4% identical (Figure 1), though they show some differences that might be relevant for their metal coordination capacity and the production of metal–MT complexes. In particular, NpeMT1 has an extra Cys in the β3 domain, while NpeMT2 has two additional histidines (His), one at the end of the β3 domain and another at β1 domain’s C-terminal end (Figure 1). These differences are shared by all Neritimorpha MT1/MT2 pairs, but not by the MTs from other gastropod clades (Figure 1). Additionally, NpeMT2′s β3 domain was independently expressed in *E. coli* cultures in order to determine the ability of this gastropod-specific domain to form metal complexes by itself. This domain was selected over NpeMT1′s β3 one due to its similarity to other gastropod β3 domains (Figure 1).

**Figure 2:**
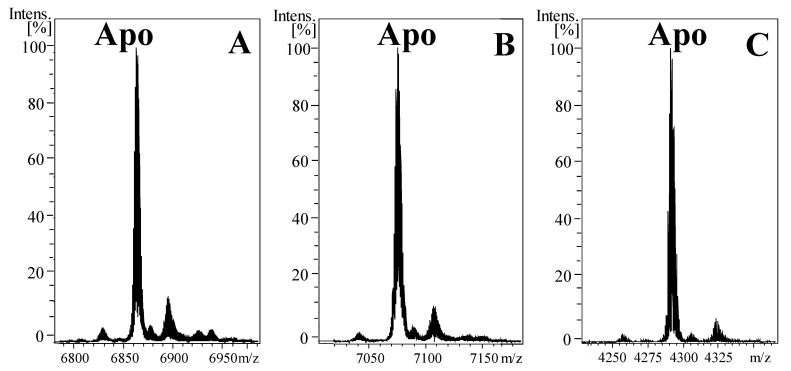
Deconvoluted ESI–MS spectra of (**A**) NpeMT1, (**B**) NpeMT2 and (**C**) β3NpeMT2 recombinantly produced in Cd-enriched *E. coli* cultures and recorded at acidic pH (pH 2.4).

Metal–protein complexes were purified from total protein extracts of *E. coli* expressing the recombinant proteins by a GST-affinity system, followed by the cleavage with thrombin of the GST tag, and a FPLC chromatography. Notice that the digestion with thrombin of the GST–MS fusion proteins resulted in the addition of two extra residues, glycine and serine, at the *N*-terminal end of the purified MTs. As shown in previous studies, these two amino acids do not interfere with the metal-binding features of recombinant MTs [32]. The FPLC fractions containing the metal–MT complexes were characterized by ESI–MS analyses (Figure 2). The experimental masses corresponding to the apo-NpeMT1 and apo-NpeMT2 (6863 and 7075 Da, respectively) after demetallation by acidification of Cd-NpeMT1 and Cd-NpeMT2 nicely match with the theoretical masses (6863.82 and 7075.93 Da). The same holds true for the synthesis of the β3NpeMT2 domain (Figure 2), whose experimental mass (4291 Da) is perfectly concordant with the expected one (4291.83 Da).

### 2.2. The Metal-Binding Abilities of NpeMT Isoforms towards Divalent Metal Ions Confirms a Cd-Selective Origin in Snail MTs

The Cd-selectivity and its habitat-related modulation are evolutionary hallmarks in MT families of gastropod clades, apparently promoted by the continuous impact of Cd through geological eras and by adaptation of gastropod lineages to different marine, terrestrial, and freshwater realms [10]. An important key to this MT versatility has been the capacity of mollusks to evolve novel metal-binding MT domains and to multiply and combine them differently in a clade-specific manner [11]. In this way, novel domains and MTs have been invented, some of them exhibiting a particularly high Cd^II^ binding capacity or a binding preference for Cu^I^ [33,34]. As demonstrated by accompanying functional studies with metal-exposed snails in vivo, the metal-selective MT isoforms are often involved in metal-specific tasks in favor of the preferred metal ion bound by the respective isoform [14,21,35]. In freshwater snails of Caenogastropoda and Heterobranchia clades, on the other hand, metal selectivity of MTs has secondarily been lost through adaptation of their hosts to freshwater environments with a lower availability of Cd^II^ [10,36].

**Figure 3:**
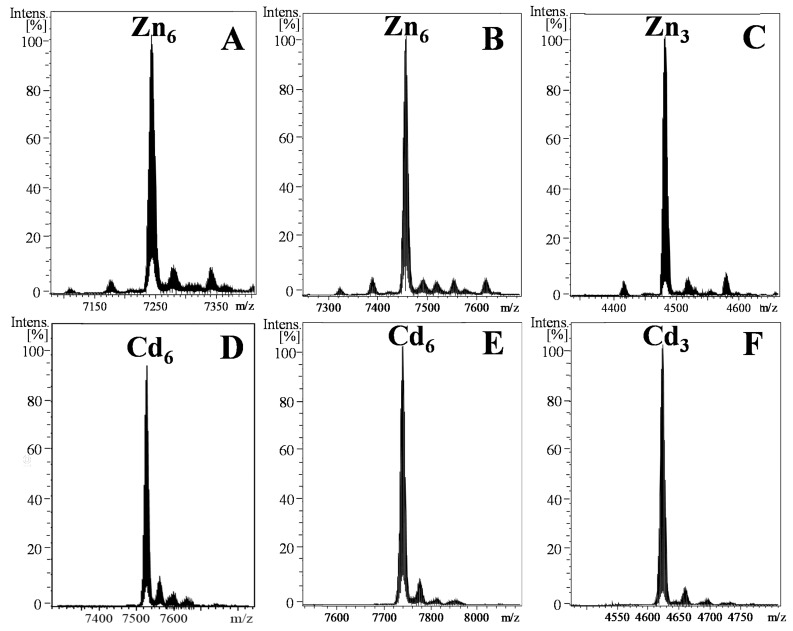
Deconvoluted ESI–MS spectra measured at pH 7.0 of the products obtained from (**A**) NpeMT1, (**B**) NpeMT2, and (**C**) β3NpeMT2 recombinant synthesis in Zn-enriched *E. coli* cultures and (**D**) NpeMT1, (**E**) NpeMT2, and (**F**) β3NpeMT2 recombinant synthesis in Cd-enriched *E. coli* cultures.

The characterization of *N. peloronta* MTs explores the metal-binding behavior of a new clade of Gastropoda, contributing with more data to complete the current insights into the evolution of snail MTs and their metal-selectivity. NpeMT1 (19 Cys) and NpeMT2 (18 Cys and 2 His) isoforms yielded species with the same metal-to-protein stoichiometries when produced in *E. coli* cultures supplemented with Zn^II^ or Cd^II^ salts (Figure 3 and Table 1), both isoforms rendering M^II^_6_–MT complexes (where M^II^ = Zn^II^ or Cd^II^). These results confirm that both proteins bind Zn^II^ and Cd^II^ metal ions with high efficiency. Interestingly, the data obtained from the independent β3NpeMT2 domain showed the formation of unique M^II^_3_–MT complexes (Figure 3 and Table 1) when the peptide is in association with the divalent metal ions. This indicates that the β3 domain can form single and structurally well-defined M^II^_3_(SCys)_9_ clusters, and therefore, that this gastropod-specific domain can be considered a functionally autonomous module. Since the full NpeMT2 protein is in association to six metal ions, our results imply that the carboxyl-terminal β1 domain also coordinates three divalent metal ions, and thereby, it is not daring to assume that the NpeMTs have a modular organization, in which each β domain forms independent M^II^_3_(SCys)_9_ clusters. Interestingly, other invertebrate MT domains, including the γ domain of patellogastropods [11], the 12C domains of tunicates [12,37], and a single metal-binding MT domain isolated by chromatographic separation methods from an earthworm [38], also show highly autonomous metal-binding abilities, confirming the modular organization of the MTs. Our data agree with recent NMR studies of the three-dimensional structure of MTs from *Littorina littorea* and *Helix pomatia*, which revealed that their MTs were organized in autonomous domains, in which 9 Cys coordinated three divalent ions [39,40], and indicated that, in contrast with what has been observed for other MTs [41,42], the additional Cys in NpeMT1 and the two extra His in NpeMT2 (Figure 1) do not increase the metal-binding capacity of these proteins. These extra residues might be likely related with the metal preference of NpeMTs (see below). 

Overall, the present study proves for the first time that the metal-selectivity towards divalent metal ions is evidently a feature that Neritimorpha MTs share with Cd-selective MTs of other gastropod clades, a question, that has so far been unresolved [10].

### 2.3. Cd Selectivity in NpeMTs Makes Way to a Gradual Transition towards Zn Specificity

**Figure 4:**
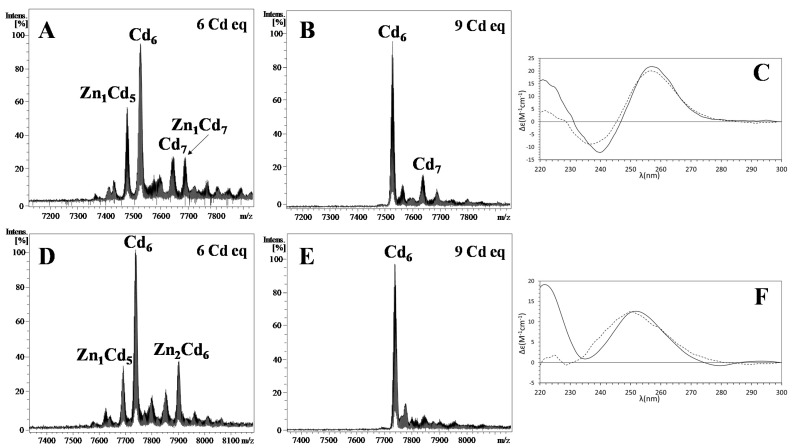
Deconvoluted ESI-MS (pH 7.0) spectra of (**A**) Zn-NpeMT1 + 6 Cd^II^ eq, (**B**) Zn-NpeMT1 + 9 Cd^II^ eq, (**D**) Zn-NpeMT2 + 6 Cd^II^ eq, (**E**) Zn-NpeMT2 + 9 Cd^II^ eq. (**C**) CD spectra of Cd-NpeMT1 (solid) and Zn-NpeMT1 + 9 Cd^II^ eq (dashed). (**F**) CD spectra of Cd-NpeMT2 (solid) and Zn-NpeMT2 + 9 Cd^II^ eq (dashed).

The Zn^II^/Cd^II^ exchange experiments provided information about the Zn-thiolate cluster lability and, thus, the NpeMTs’ metal-binding preference, by measuring how hastily Zn^II^ can be replaced by Cd^II^. These metal replacement experiments evolved parallelly in NpeMTs. As observed in the final stages of the titration, monitored by ESI–MS and CD spectra (Figure 4), both NpeMTs achieve the formation of Cd_6_-MT complexes, requiring the addition of 6 Cd^II^ equivalents. It is not until the addition of 9 Cd^II^ equivalents that all trace of Zn^II^ fully disappears from the metal–MT complexes. 

The chromophores obtained at the final stage of both titrations render CD spectra that nicely reproduce those of their respective recombinantly produced samples (Figure 4C,F), denoting the in vitro formation of isostructural complexes.. These results suggest that the metal-binding features of the two NpeMTs fulfill to a large degree the characteristics of the currently known M^II^-thioneins, a group that in our classification scheme did so far include Zn- and Cd-selective MTs [16]. Interestingly, the ESI–MS spectra revealed the in vitro presence of Cd^II^_6_–MT complexes for both isoforms but also the presence of minor Cd^II^_7_–MT species in the case of NpeMT1 (Figure 4B). This difference in the products of the metal exchange experiment suggests a slight variation in the coordinating abilities between both isoforms, probably as a result of the functional divergence of the NpeMTs by a process of the neo- or sub-functionalization from an ancestral Cd-selective snail form. Most likely, the folding pathway of these NpeMTs and, thus, their cluster formation, differ from each other, probably as a result of the fact that they possess some non-shared particular amino acids (see Section 1). This variation does not imply crucial dissimilarities in their metal-binding abilities towards divalent metal ions per se, but causes a marked distinction when coordinating Cu^I^ ions (see below). Overall, the fact that the metal-exchange experiments led to final NpeMT complexes that completely resemble the recombinantly produced primary complexes proves that these complexes are energetically favored and confirms that *Nerita* *peloronta* possesses two M^II^-selective MTs, as predicted in a previous work [10].

### 2.4. NpeMT Isoforms Reveal Important Differences in Their Cu^I^ Binding Features

**Figure 5:**
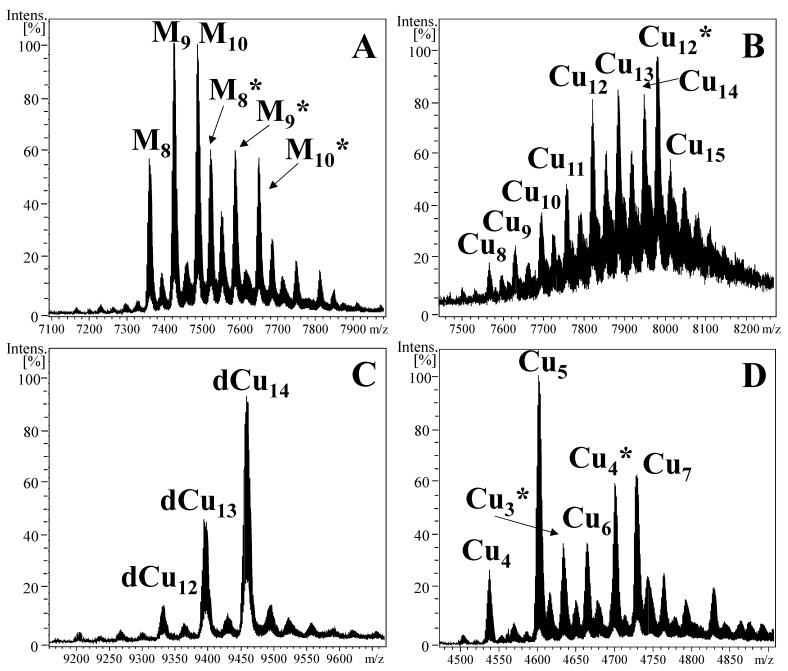
Deconvoluted ESI–MS (pH 7.0) spectra of (**A**) Cu-NpeMT1, (**B**) Cu–NpeMT2, (**C**) Cu-β3NpeMT2 type 1, and (**D**) type 2. M in panel A stands for (Zn + Cu)–NpeMT1 complexes. Species with * correspond to metal complexes formed by glycosylated-MTs and species with d- correspond to dimeric complexes.

In this section, it will be shown that the differences in the metal-binding features of the two NpeMTs after recombinant production under Cu supplementation provide important clues to better understand their differential M^II^-thionein behavior. In fact, the most significant difference between the two NpeMTs lies on their Cu^I^ metal-binding abilities. The products obtained from the Cu^II^-enriched cultures show that while NpeMT1 renders a mixture of heteronuclear Zn,Cu–MT complexes ranging from M_8_ to M_10_ (where M is the combination of Zn^II^ and Cu^I^ ions), NpeMT2 yields a mixture of homonuclear Cu-MT complexes ranging from Cu_8_ to Cu_15_ (Table 1, Figure 5).

The dissimilarities between the amino acid sequences of NpeMT1 and NpeMT2 (see Figure 1) partially allow to explain some differences in their respective Cu^I^-binding features. These variations lie in the nature and number of the amino acids involved in the metal coordination (i.e., Cys and potentially His). Interestingly, the presence of His residues in Cu-specific gastropod MTs, such as HpCuMT or CaCuMT, has been proved to aid in the binding and releasing of Cu^I^ ions [43], suggesting that the presence of His could be a key element for the metal-binding ability of NpeMT2. The detection of Zn^II^ ions in the Cu-enriched synthesis of NpeMT1, however, denotes the incapability of this isoform to form stable homometallic clusters with Cu^I^ and clearly proves that its metal-binding abilities are far from a Cu-thionein and, in fact, denotes that NpeMT1 exhibits common traits of a genuine Zn-thionein [19]. On the other side, despite the homonuclear nature of the Cu–NpeMT2 complexes, the fact that the protein does not give rise to a unique and therefore thermodynamically favored Cu–NpeMT2 species unambiguously proves that this isoform neither behaves as a true Cu-thionein.

Interestingly, the synthesis of the single β3NpeMT2 domain under Cu-supplementation also rendered a mixture of homometallic Cu complexes that were separated by FPLC as dimeric (Figure 5C) and monomeric species (Figure 5D). This clearly confirms that the β3NpeMT2 domain cannot form a unique independent metal-cluster with Cu^I^ as it does for divalent metal ions, suggesting a different distribution of Zn^II^/Cd^II^ and Cu^I^ between the two β domains that conform NpeMT2. The presence of dimers in the sample also denotes that the β3NpeMT2 peptide cannot efficiently form Cu clusters by itself, freeing some Cys residues that interact, via Cys–metal–Cys bridges, with Cys residues of other complexes, gaining in thermodynamic stability. Importantly, β3NpeMT2 metal-binding preferences remain the same as those of NpeMT2, rendering exclusively homometallic Cu-MT species. It contains nine Cys and one His residues, thus maintaining the biochemical characteristics of the full protein. This is most likely the reason why this domain reproduces the same metal-binding abilities than the entire protein.

Another point to be mentioned is that some glycosylated species are detected in the Cu-NpeMTs productions (marked with asterisk (*) in the corresponding spectra of Figure 5). The intensity of the ESI–MS peaks denote that these glycosylated species are important species in these samples. However, this phenomenon is out of the scope of this paper and an on-going publication will thoroughly detail the causes and consequences of glycosylation on MTs.

**Figure 6:**
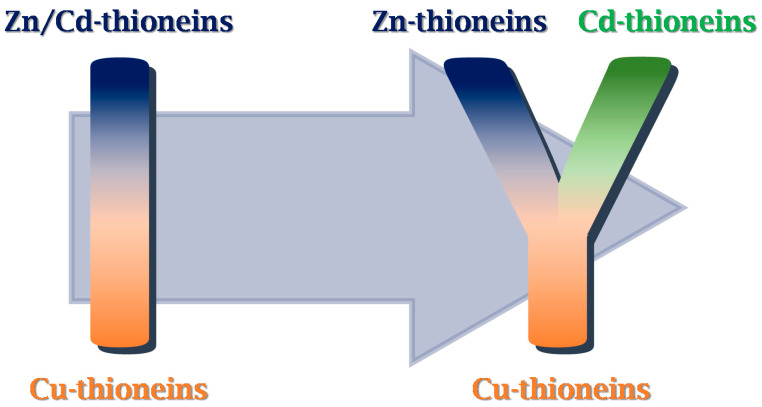
Scheme of the new MT classification proposal that considers the differentiation between genuine Zn- and Cd-thioneins, replacing an “I” disposition to a “Y” setting.

In summary, none of the two NpeMT isoforms shows a strong metal-specificity towards Cu^I^ ions, which means that they are not Cu-thioneins. However, the samples obtained from the Cu-productions have revealed significant differences—homometallic versus heterometallic complexes—between the metal-binding abilities of the two NpeMTs. This differential character in NpeMTs with regard to Cu binding sets a nice example (from a chemical point of view) of how Zn/Cd-thioneins can discriminate in their binding behavior between those divalent metal ions (Cd^II^ and Zn^II^). Altogether, this opens a window for a modification of the current classification of MTs based on their metal-specificity [19], from a dichotomic classification of Zn/Cd(M^II^)- versus Cu(M^I^)-thioneins (an “I” setting) towards a three band classification of Zn-, Cd-, and Cu-thioneins (a “Y” setting), splitting the previous M^II^-thioneins into Zn- and Cd-thioneins (Figure 6).

### 2.5. NpeMT Isoforms Provide the Key to Set Up a Novel MT Classification Scheme

The accepted idea that MTs’ three-dimensional structure is mainly dictated by the kind of metal ion which they are coordinated to [44,45] and the fact that the structure of the protein determines its chemical functionality [46], led to the postulation that the metal ion predominantly associated to each MT is indicative of its particular biological function. Bearing this in mind, a classification of MTs sorted by their metal-binding abilities was proposed by our research group as a useful tool to infer the possible biological function of novel MTs [19]. This classification of MTs has so far covered the needs for the actual scene in the MTs’ field. Now, however, the results of the present study regarding the metal-binding abilities of NpeMTs, as well as the metal-binding abilities found in other MTs from recent studies [10,12,37,47], evidence the necessity of updating the current classification of MTs. The new classification we here propose considers that “genuine Zn-thioneins” render heterometallic Zn,Cu-MT complexes when synthesized in cultures supplemented with Cu^I^, using Zn^ÌI^ as structural ions and stabilizing the metal cluster, whereas “genuine Cd-thioneins” (introducing a new term in MT classification) render a mixture of homometallic low-structured Cu^I^-MT species, not requiring Zn^II^ to form stable complexes. NpeMT1 represents, therefore, a genuine Zn-thionein (Figure 5A), whereas NpeMT2 is a genuine Cd-thionein (Figure 5B). Noteworthy, the Cu^I^-binding abilities of NpeMT2 resemble those recently found for several Cd-selective MTs of diverse marine species [10,12,37,47]. This supports the idea that the ancestral MTs might have arisen from a detoxification system against toxic metals such as Cd, and that this was later co-opted for homeostatic functions of essential metals such as Zn or Cu [11,12].

The differential behavior found in the Cu^I^-binding abilities between NpeMT1 and NpeMT2 resulted to be a good starting point allowing to differentiate between “genuine Zn-thioneins” and “genuine Cd-thioneins”. This novel gradation scheme among three distinct types of “genuine MTs” that contrast with the actual concept of a dichotomic classification between “genuine Zn/Cd-thioneins” and “genuine Cu-thioneins” will be proposed and explained in more detail in another work (in preparation). There, we will assemble all the known Cd-thionein examples and that provide more evidences for additional metal-binding features supporting this new and more differentiated classification scheme for this big family of metalloproteins (Figure 6).

### 2.6. Biological Implications

NpeMT1 and NpMT2 and the C-terminal metal-binding domain β3NpeMT2 of *N. peloronta* are the first MTs analyzed from a snail of the gastropod clade of Neritimorpha. With a number of about 1500 known species [48], Neritimorpha form a rather small clade with an astonishingly high diversity in terms of morphology and adaptation to extreme marine environments, including—apart from *N. peloronta*—a numerous group of neritid snails thriving in the intertidal splash zones of tropical regions [26]. The results of our present investigation prove that Cd-selective MTs are apparently also present in the gastropod clade of Neritimorpha, a finding that has so far not been known [10], although NpeMT1, in particular, exhibits some features that suggest a beginning transition from a genuine Cd-thionein to a genuine Zn-thionein. In this context, it must be noted that several studies have demonstrated the capacity of neritid species to accumulate metals in their soft tissues, sometimes in dependence of environmental exposure. In most of these studies, it was found that Zn and Cu exhibited the highest concentrations in snail soft tissues [30,31,49,50], in which a high correlation was found between Zn and Cd accumulation [29]. Overall, however, Cd seems to accumulate at higher rates in shells and opercula than in soft tissues of neritid species [50]. It is assumed that among the so-called “soft tissues” of these snails, the most important organ for metal accumulation is probably the digestive gland, as generally demonstrated for other gastropod snails, too [51].

Interestingly, these two isoforms with quite relevant differences in their amino acid sequence (i.e., they are only 42% identical in non-coordinating residues, and NpeMT2 contains His residues in contrast to NpeMT1) have been maintained during the evolution of this species. In fact, also the other species within this clade share MT isoforms homologous to NpeMT1 and NpeMT2, suggesting that Neritimorpha MTs have undergone a process of functional divergence in a lineage-specific manner, and that recently both isoforms are relevant and exert important biological functions. However, from these scanty metal accumulation studies and our own findings about metal-selectivity of *N. peloronta* MTs, it would be premature to infer a major Zn- or Cd-related biological roles for NpeMT1 and/or NpeMT2. Although it is tempting to hypothesize such roles, it is clear that only additional in-depth research about the function of NpeMTs and their impact on metal distribution in vivo, combined with physiological studies, could solve this question. 

What the present study shows, however, is the fact that from a bioinorganic-chemical point of view, the MTs of *N. peloronta* mark a crossroads position between purely Cd-selective and Zn-selective MTs, with probably relevant consequences for their different biological functions in the organism. This has also implications for the classification of gastropod MTs [19] in general, and challenges to consider the possibility that from a chemical point of view, MTs can develop pure selectivity preferences for either of the three metal ions, Cd^II^, Zn^II^, and Cu^I^.

## 3. Materials and Methods

### 3.1. Cloning, Production, and Purification of Recombinant Metal–MT Complexes

Synthetic cDNAs codifying NpeMT1, NpeMT2, and β3NpeMT2 (from amino acid 1 to 39) were provided by Synbio Technologies (Monmouth Junction, NJ, USA), cloned as GST-fusion products in the pGEX-4T-1 expression vector (GE Healthcare, Chicago, IL, USA) and transformed in *Escherichia coli* BL21 (protease-deficient strain) for heterologous expression. For protein production, 500 mL of Luria-Bertani (LB) medium with 100 μg mL^−1^ ampicillin was inoculated with *E. coli* BL21 cells transformed with the corresponding recombinant plasmids. After overnight growth at 37 °C/250 rpm, the cultures were used to inoculate 5 L of fresh LB-100 μg mL^−1^ ampicillin medium. Gene expression was induced with 100 μM isopropyl-β-D-thiogalactopyranoside (IPTG) for 3 h (h). After the first 30 min of induction, cultures were supplemented with ZnCl_2_ (300 μM), CdCl_2_ (300 μM), or CuSO_4_ (500 μM) in order to generate metal–MT complexes. Cells were harvested by centrifugation for 5 min at 9100× *g* (7700 rpm), and bacterial pellets were suspended in 125 mL of ice-cold phosphate-buffered saline (PBS: 1.4 M NaCl, 27 mM KCl, 101 mM Na_2_HPO4, 18 mM KH_2_PO_4_, and 0.5% *v/v* β-mercaptoethanol). Resuspended cells were sonicated (Sonifier Ultrasonic Cell Disruptor) 8 min at voltage 6 with pulses of 0.6 s, and then centrifuged for 40 min at 17,200× *g* (12,000 rpm) and 4 °C.

Soluble protein extracts containing GST–MS fusion proteins were incubated with glutathione sepharose beads (GE Healthcare) for 1 h at room temperature with gentle rotation. GST–MS fusion proteins bound to the sepharose beads were washed with 30 mL of cold 1×PBS bubbled with argon to prevent oxidation. After three washes, GST–MS fusion proteins were digested with thrombin (SERVA Electrophoresis GmbH Heidelberg, Germany, 25 U L^−1^ of culture) overnight at 17 °C, thus enabling separation of the metal–MT complexes from the GST that remained bound to the sepharose matrix. The eluted metal–MT complexes were concentrated with a 3 kDa Centripep Low Concentrator (Amicon, Merck-Millipore, Darmstadt, Germany), and fractionated on a Superdex-75 FPLC column (GE Healthcare) equilibrated with 20 mM Tris-HCl, pH 7.0. The protein-containing fractions, identified by their absorbance at 254 nm, were pooled and stored at −80 °C until use.

### 3.2. Characterization of the Metal-NpeMT1 and Metal-NpeMT2 Complexes

The recombinant proteins obtained as metal-NpeMTs preparations were recovered from *E. coli* culture media supplemented with Zn^II^, Cd^II^, or Cu^II^ salts. Sulphur content measurement in samples by means of Inductively Coupled Plasma Atomic Emission Spectrometer (ICP–AES) allowed the determination of the protein concentrations, as well as the metal-to-protein stoichiometry [52]. The technique was performed in an Optima 4300DV (Perkin-Elmer, Waltham, MA, USA) spectrometer by measuring S at 182.04 nm, Zn at 213.85 nm, Cd at 228.80 nm, and Cu at 324.75 nm.

All samples MW determinations were obtained by means of Electrospray Ionization Time-of-Flight Mass Spectrometry (ESI–TOF MS) using a Micro TOF-Q instrument (Bruker Daltonics, Bremen, Germany) interfaced to a Series 1200 HPLC Agilent pump and controlled by Compass Software. ESI-L Low Concentration Tuning Mix (Agilent Technologies, Santa Clara, CA, USA) was used as calibrator. A 5:95 mixture of acetonitrile:ammonium acetate (15 mM) and a 5:95 mixture of acetonitrile:formic acid solution was used as running buffer for neutral (pH 7.0) and acidic (pH 2.4) conditions, respectively. Instrument conditions were as follows: 10–45 µL of sample solution were injected through a polyether heteroketone (PEEK) tube (0.5–1.5 m, 0.18 mm i.d.) at 25–50 µL·min^−1^, applying a capillary counter-electrode voltage of 3.5–5.5 kV; a dry temperature of 90–110 °C; dry gas at 6 L min^−1^; and spectra collection range of 800–3000 *m/z*. The acid pH causes the release of Zn^II^ and Cd^II^, but Cu^I^ remains complexed to the protein. All experimental mass values were calculated as previously described in [53].

Circular dichroism (CD) measurements were recorded in a Jasco spectropolarimeter (Model J-715, JASCO, Groß-Umstadt, Germany), interfaced to a computer (J700 software, JASCO, Groß-Umstadt, Germany) and keeping the temperature at 25 °C using a Peltier PTC-351S equipment (TE Technology, Traverse City, MI, USA). UV-vis spectroscopy was performed in a HP-8453 Diode array UV-Visible spectrophotometer (Hewlett-Packard, Palo Alto, CA, USA). Quartz cuvettes (1-cm) were employed for spectra recording and all spectra obtained from both techniques was processed with GRAMS 32 Software (GRAMS/AI v.7.02; Thermo Scientific, Walthman, MA, USA).

### 3.3. Metal-Protein Binding Studies

The so-called in vitro Cd^II^-MT samples were obtained by exchanging the Zn^II^ ions of the corresponding biosynthesized Zn^II^-MT preparations by adding molar equivalents of a CdCl_2_ solution, as described elsewhere [54]. The experiments were performed at pH 7.0, the solutions were bubbled with argon after every metal addition and aliquots were sampled to perform ESI–MS studies. The experiments were monitored by performing CD and UV-vis measurements at each metal addition.

## Figures and Tables

**Table 1:** Protein concentration and metal-to-protein ratio of the recombinant NpeMT1, NpeMT2, and β3NpeMT2 purified from Zn^II^-, Cd^II^-, and Cu^II^-enriched *E. coli* cultures.

Isoform	Supplemented Metal Ion ^a^	Protein Concentration (10^−4^ M)	Metal-to-Protein Ratio ^b^
NpeMT1 **(19 Cys)**	Zn^II^	2.1	5.9 Zn
	Cd^II^	1.0	5.5 Cd
	Cu^II^	1.0	1.9 Zn; 7.1 Cu
NpeMT2 **(18 Cys + 2 His)**	Zn^II^	2.9	5.9 Zn
	Cd^II^	1.4	5.5 Cd
	Cu^II^	0.3	12.0 Cu
β3NpeMT2 **(9 Cys + 1 His)**	Zn^II^	3.7	2.8 Zn
	Cd^II^	3.7	2.6 Cd
	Cu^II^ (Dimers)	0.6	5.3 Cu
	Cu^II^ (Monomers)	0.6	5.6 Cu

^a^ β3NpeMT2 synthesis under Cu-supplementation rendered two families of metallated species, which were separated through FPLC, characterized separately and that corresponded to the dimeric and monomeric complexes of this construction. ^b^ Metal-to-protein ratios calculated from Zn, Cd, and Cu concentrations obtained by ICP–AES. All three metals were measured in all cases but only those results different than zero are shown.

## Data Availability

Not applicable.
